# Lactobacillus mediates the expression of NPC1L1, CYP7A1, and ABCG5 genes to regulate cholesterol

**DOI:** 10.1002/fsn3.2600

**Published:** 2021-09-30

**Authors:** Kaihui Cao, Kaiping Zhang, Muran Ma, Junjie Ma, Jianjun Tian, Ye Jin

**Affiliations:** ^1^ College of Food Science and Technology Inner Mongolia Agricultural University Hohhot China; ^2^ Department of Cooking & Food Processing Inner Mongolia Business and Trade Vocational College Hohhot China

**Keywords:** cholesterol, lactobacillus, mechanism, regulation

## Abstract

Hypercholesterolemia is the main cause of cardiovascular disease worldwide, and the regulation of cholesterol homeostasis is essential for human health. Lactobacillus is present in large quantities in the human intestine. As the normal flora in the gut, lactobacillus plays an important role in regulating metabolism in the human body. Lactobacillus can regulate the cholesterol content by regulating the expression of genes involved in cholesterol synthesis, metabolism, and absorption. This article reviews the biological effects and mechanisms of lactobacillus that mediate the expression of NPC1L1, CYP7A1, ABCG5, ABCG8, and other genes to inhibit cholesterol absorption, and discusses the mechanism of reducing cholesterol by lactobacillus in cells in vitro, to provide a theoretical basis for the development and utilization of lactobacillus resources.

## Graphical abstract

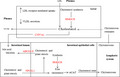

## 1 INTRODUCTION

With the development of the social economy and the improvement of living standards, coronary heart disease (CHD) has become the most important cause of death globally (Fabian et al., 2016); the most common form of coronary heart disease is coronary artery disease (CAD). There is a positive correlation between elevated serum total cholesterol levels (mainly low‐density lipoprotein cholesterol (LDL‐C)) and the risk of coronary heart disease. And there is a linear relationship between the increase in LDL‐C concentration and the relative risk of CAD. Through a large number of articles, the effect of lactobacillus on the content of LDL‐C in serum was analyzed. Studies have found that every 1 mmol increase in serum cholesterol levels increases the risk of CHD by about 35% and coronary heart disease‐related mortality by 45%; every 1% decrease in serum cholesterol levels reduces the risk of CHD by 2%–3% (Liong & Shah, 2005). Moreover, statins are effective in treating hypercholesterolemia; however, such drugs are expensive and have harmful side effects (Stancu & Sima, 2001). People prefer non‐drug and healthy methods to control serum cholesterol levels.

Lactobacillus is an abundant microorganisms in the human body. Lactobacillus not only has nutritional functions, but also has other functions, such as antibacterial, antitumor, and immune regulatory activity. In addition, studies have found that lactobacillus can reduce serum cholesterol (Tian et al., 2019). Therefore, lactobacilli have attracted the attention of researchers, and the mechanism of lowering cholesterol has also been studied extensively.

## 2 STUDY ON CHOLESTEROL REGULATION BY LACTOBACILLUS

Several studies have shown that lactobacillus can reduce the cholesterol content. The initial determination of the cholesterol‐lowering ability of lactobacillus only stayed in the ability to reduce cholesterol content in the culture medium, through coprecipitation (Klaver & van der Meer, 1993), assimilation, and absorption (Pereira & Gibson, 2002). Studies have found that lactobacillus can produce bile salt hydrolase (BSH), which can catalyze the hydrolysis of bile salt into amino acids and free bile acids. Free bile salt forms a complex with cholesterol. The precipitation of the complex reduces cholesterol content (Griffiths et al., 2011). Ren Dayong et al. screened lactobacilli with strong cholesterol‐lowering ability from traditional fermented foods in Northeast China and found that the selected lactic acid bacteria can reduce cholesterol content through membrane adsorption, coprecipitation, bile salt hydrolysis enzyme, etc., of which the main effect is bile salt hydrolysis enzyme (Ren et al., 2019). At the same time, the medium environment can affect the precipitation of cholesterol by lactobacillus. Young et al. found that the dormant and growing cells of *Bifidobacterium* can precipitate and remove cholesterol in the presence of bile salts and pH <5.4 (Young et al., 1988). Dao Dong Pan et al. showed that *L*. *fermentum* SM‐7 can coprecipitate and absorb 38.5% of the cholesterol in the culture medium, and the coprecipitation of cholesterol and bile acid increased rapidly when pH <6 (Pan et al., 2011). Assimilation and absorption involve cholesterol absorption by lactobacillus through the cell wall, cell membrane, and cytoplasm in an anaerobic environment. Anila et al. found that in broth containing bile salts, the removal rate of total cholesterol (TC) by lactobacillus was significantly higher than that in the non‐bile salt group (Anila et al., 2016). Moreover, the assimilation effect of growing cells on cholesterol was significantly higher than that of resting cells and dead cells, whereas no significant difference between dormant cells and dead cells was observed for cholesterol removal and assimilation (*p* < .05); the ability of lactobacillus to absorb cholesterol during death and dormancy indicates that cholesterol may also be eliminated by binding to the cell surface. Lim et al. evaluated the cholesterol‐lowering ability of *Lactobacillus* LAB4 and *L. plantarum* LAB12 in the growth medium (Lim et al., 2017); cholesterol reduction rate of both lactobacilli was greater than 98%; Nile red staining revealed that lactobacillus absorbed cholesterol directly.

Thereafter, lactobacillus with the ability to lower cholesterol began to be used in animal experiments, and the mechanism of regulating cholesterol content by lactobacillus was studied in vivo. Gilliland et al. discovered that under anaerobic conditions, *L. acidophilus* (isolated from pig feces) can reduce the cholesterol content in a cholesterol medium containing bile salts (Gilliland et al., 1985). The content of bile salts in the medium is different, and the amount of cholesterol reduction is also different. Moreover, serum cholesterol content in pigs fed on *L. acidophilus* did not increase significantly compared with pigs on a normal diet. Yadav and other studies found that when rats on a high cholesterol diet were fed fermented milk containing *L*. *fermentum* for 90 days, serum TC, low‐density lipoprotein cholesterol, triglycerides, very low‐density lipoprotein cholesterol, atherosclerosis index, coronary artery risk index, liver lipid, and lipid peroxidation degree were reduced significantly (*p* < .001), and the mRNA expression of inflammatory cytokines, namely TNF‐α and IL‐6, was found significantly (*p* < .001) higher in the cholesterol‐enriched diet group compared to the group fed fermented milk containing *L*. *fermentum* in the liver. Research suggests that *L*. *fermentum* can be used as a potential probiotic for treating hypercholesterolemia (Yadav et al., 2018). Park et al. reached the same conclusion on lactobacillus isolated from kimchi and found that *Leuconostoc mesenteroides* subsp. KDK411 can improve hypercholesterolemia in rats by absorbing and excreting cholesterol (Park et al., 2018). Yin Boxing et al. found that the contents of triglyceride, TC, and low‐density lipoprotein cholesterol in the serum of rats could be reduced by adding lactobacillus into rat diet, and the abundance and diversity of intestinal flora in rats were significantly increased (Yin et al., 2019). In vivo experiments showed that lactobacillus could regulate the expression of cholesterol metabolism related genes. By adding *L. paracasei* TD3 to rat feed, Dehkohneh et al. found that the intake of *L. paracasei* TD3 can significantly reduce serum cholesterol level in rats, and the expression of the key gene of cholesterol metabolism, namely 3‐hydroxy‐3‐methylglutaryl CoA reductase (HMGCR), and cytochrome P450 7A1 (CYP7A1) decreased significantly (Dehkohneh et al., 2019). Guo Lidong et al. studied the BSH activity and cholesterol‐lowering effect of *L. plantarum* KLDS 1.0344 and found that BSH activity varies with the content of bile salt. Moreover, the KLDS 1.0344 strain regulated the expression of cholesterol metabolism related genes, including the downregulation of HMGCR and farnesine X receptor (FXR) in the liver and NPC1L1 in the intestine and the upregulation of LXRα, ABCG5, ABCG8, and ABCA1 in the intestine and CYP7A1 in the liver (Guo et al., 2019).

Research on cholesterol‐reducing lactobacillus has developed rapidly. The initial research focuses on the isolation and screening of lactobacillus with cholesterol‐lowering ability. Recently, it has focused on the study of the cholesterol‐lowering mechanism of lactobacillus, and research on its mechanism is warranted.

## 3 LACTOBACILLUS MEDIATES THE EXPRESSION OF RELATED GENES TO REGULATE CHOLESTEROL

**FIGURE 1:**
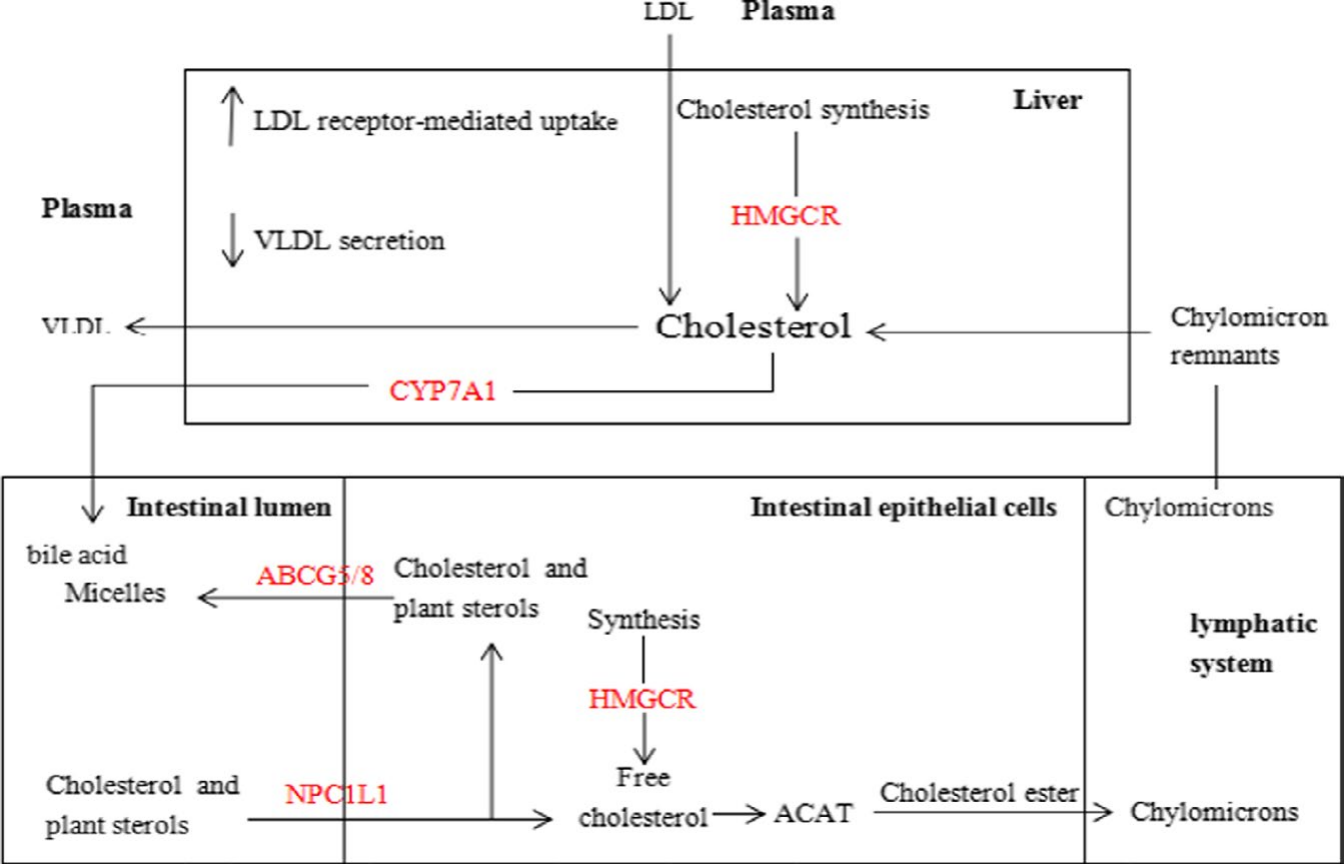
Cholesterol metabolism in vivo

Cholesterol is an important component of the human body. It is not only an important component of biofilms, but also the precursor of substances, such as vitamin D and bile acid (Liong & Shah, 2005). Cholesterol metabolism in the body is shown in Figure 1 (Morgan et al., 2017).

The balance of cholesterol level in the body is regulated by four aspects, namely synthesis, catabolism, absorption, and transportation of cholesterol. After cholesterol is synthesized in the liver by 3‐hydroxy‐3‐methylglutaryl CoA reductase (HMGCR), a part of it is decomposed into bile acid by cholesterol 7α‐hydroxylase (CYP7A1). After entering the intestine, bile acid can synthesize bile salt micelles with cholesterol. The absorption of bile salt micelles in intestinal epithelial cells is mainly controlled by Niemann–Pick C1‐like 1 (NPC1L1). Free cholesterol enters the intestinal lumen through the ATP‐binding cassette transporter family members G5 and G8 (ABCG5/G8) and is excreted in feces. Some key control factors regulating cholesterol metabolism are shown in Table 1. Therefore, the mechanism of cholesterol‐lowering by lactobacillus will be demonstrated from three aspects.

**TABLE 1:** Key control factors of cholesterol metabolism

Key control factors	Existing parts	Main regulating effect	Action direction of lactobacillus	Reference
HMGCR	Endoplasmic reticulum	Regulate cholesterol synthesis	Suppressing the expression of this protein can hinder cholesterol synthesis	(DeBose‐Boyd & Russell, 2008; Roitelman, 1992)
AMPK	Mammalian cell extracts	Participate in lipid metabolism through phosphorylation	Promote phosphorylation of the protein and inhibit HMGCR expression and then hinder cholesterol synthesis	(Srivastava et al., 2012)
CYP7A1	Liver	Regulate the breakdown of cholesterol into bile acids	Up‐regulating the expression of this protein will promote cholesterol conversion	(Chambers et al., 2019; Pullinger et al., 2002)
NPC1L1	Intestinal epithelial cells; liver	Regulate the absorption of cholesterol in intestine	Down‐regulating the expression of this protein will inhibit the intestinal absorption of cholesterol	(Iyer et al., 2005; Wang et al, 2012)
ABCG5/G8	Hepatocytes; bile duct cells; gallbladder epithelial cells; intestinal epithelial cells	Regulate cholesterol metabolism in intestine	Up‐regulating the expression of this protein will promote the intestinal metabolism of cholesterol	(Yu et al., 2002)
LXR	Liver; adipose tissue; small intestine	After activation, it can regulate bile acid synthesis and metabolism/excretion, cholesterol biosynthesis, and cholesterol absorption/excretion in the intestine	Activating the protein can promote cholesterol efflux and inhibit cholesterol absorption	(van der Veen et al., 2005)
PPAR	Colonic; small intestinal mucosa	Regulate the expression of genes related to lipid metabolism, and maintain lipid homeostasis	Up‐regulating the expression of this protein can inhibit the expression of NPC1L1 and then inhibit the intestinal absorption of cholesterol	(Wan et al., 2013; Yoon et al., 2011)
SREBP	Liver	Regulate the absorption of cholesterol in liver	Up‐regulating the expression of this protein can inhibit the liver absorption of cholesterol	(Horton et al., 2002)

### 3.1 Lactobacillus‐mediated AMPK phosphorylation affects the rate‐limiting enzyme HMGCR for cholesterol synthesis

**FIGURE 2:**
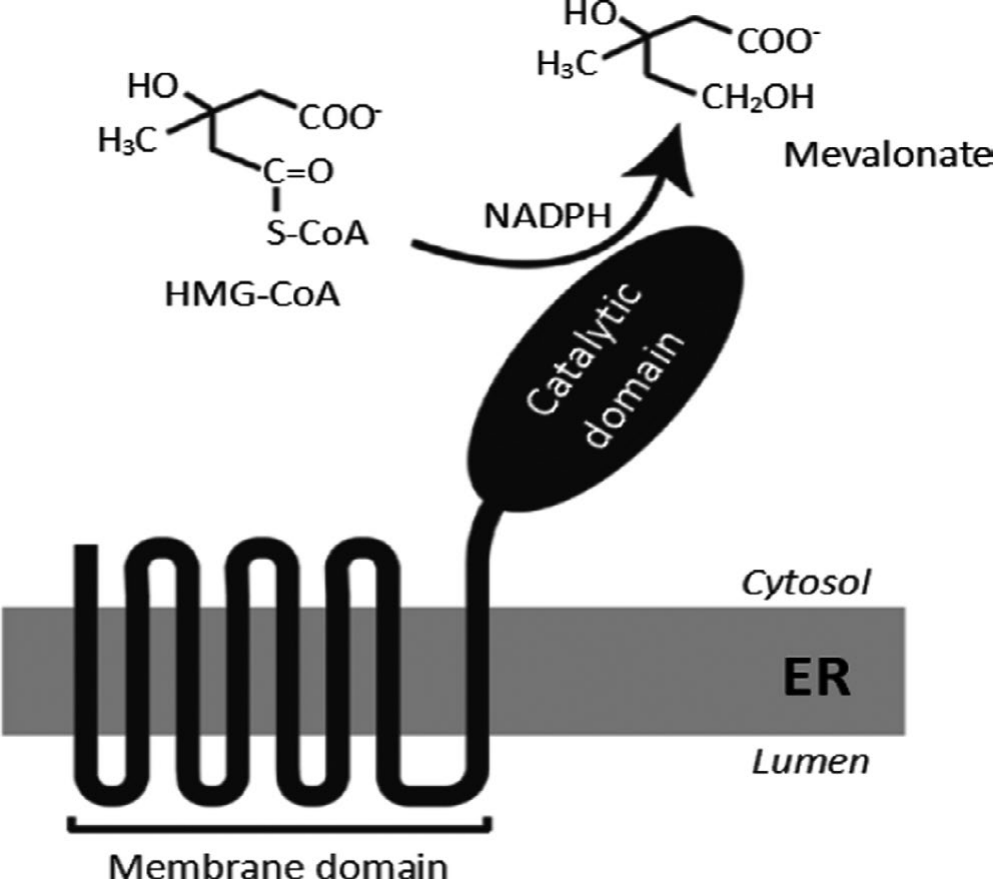
Structure of HMGCR. A hydrophobic N‐terminal domain with eight membrane‐spanning segments that anchor the protein to ER membranes, and a hydrophilic C‐terminal domain that projects into the cytosol and exhibits all of the enzyme's catalytic activity

Human cholesterol is mainly biosynthesized in the liver by mevalonic acid. In addition, cholesterol is synthesized in the intestine and adrenal glands (Tahri et al., 1996). HMGCR reduces 3‐hydroxy‐3‐methylglutaryl coenzyme A (HMG CoA) to mevalonate, which is the precursor of cholesterol. HMGCR is the rate‐limiting enzyme in cholesterol synthesis and is therefore the focus of the regulation of cholesterol synthesis (DeBose‐Boyd & Russell, 2008). HMGCR located in endoplasmic reticulum in mammals, composed of 888 amino acids, is divided into two domains. The C‐terminal consists of 548 amino acids, and the C‐terminal extends into the cytoplasm and possesses enzyme activity (Roitelman, 1992), shown in Figure 2.

Adenosine 5'‐monophosphate (AMP)‐activated protein kinase (AMPK) is an upstream kinase of cholesterol metabolizing enzymes, such as HMGCR. AMPK can control the activity of key metabolic regulators and transcription factors involved in the control of glucose and lipid metabolism through direct phosphorylation (Srivastava et al., 2012). Lew et al. found that 30% of the cell‐free supernatant of *L. plantarum* DR7 can reduce the accumulation of cholesterol in HepG2 and HT‐29 cells and reduce the mRNA expression of HMGCR in HepG2 cells. In the presence of AMPK inhibitors, the expression of HMGCR also decreased, indicating that *L. plantarum* DR7 plays a role through the AMPK pathway and significantly increased the phosphorylation of AMPK, resulting in a decrease in HMGCR expression (Lew et al., 2018). Pei‐Gee Yap et al. found that the concentration of allantoin in metabolites increased significantly in rats fed with *L. plantarum* DR7 and *L*. *reuteri* 85 for 13 days, and allantoin promoted AMPK activation by reducing the free energy of substrate binding. In addition, allantoin inhibited cholesterol biosynthesis by inducing enzymes to inhibit, occupy, or block putative binding sites, such as HMGCR, mevalonate kinase (MVK), and octosterol demethylase (LDM) non‐spontaneous substrate binding (Yap et al., 2020). Chen et al. identified an NF‐κB binding site at nt‐265 bp of the HMGCR gene. This site has the main regulatory element for cholesterol synthesis in HepG2 cells (Chen et al., 2016). Although the NF‐κB subunit is not directly phosphorylated by AMPK, the inhibition of NF‐κB signaling is mediated by several downstream targets of AMPK. In addition, NF‐κB is essential for lactobacillus mediated HMGCR gene regulation, and the NF‐κB activation pathway may be a potential therapeutic target for HMGCR signaling.

### 3.2 Lactobacillus mediates the effect of CYP7A1 on cholesterol catabolism

The synthesis of bile acids plays a vital role in maintaining cholesterol homeostasis in mammals. CYP7A1 expression is liver specific that encodes cholesterol 7α‐hydroxylase, which catalyzes the first step of cholesterol catabolism and bile acid synthesis (Pullinger et al., 2002). Increased expression of CYP7A1 can increase cholesterol excretion. More than half of the cholesterol in the human body is converted into bile acid through CYP7A1 and then excreted. During reverse cholesterol transport in surrounding tissues of the liver, cholesterol is mainly transferred to high‐density lipoprotein particles through the rate‐limiting enzyme CYP7A1 and then returns to the liver to be converted into bile acids (Chambers et al., 2019). Guo Lidong et al. found that compared with the high‐cholesterol diet control group, more CYP7A1 protein expression was observed in the liver of rats fed with *L. plantarum* KLDS 1.0344. The cholesterol‐lowering effect of 1.0344 may be mediated by upregulating CYP7A1 expression, which accelerates the conversion of liver cholesterol to bile acids, thereby releasing more fecal bile acids (Guo et al., 2019). Liver X receptor α (LXRα) and FXR are positive and negative regulators of target genes involved in regulating lipid balance in vivo, respectively. In vivo, FXR is more efficient than LXRα in inhibiting CYP7A1 mRNA expression (Ando et al., 2005). Tianming Qu et al. showed that lactic acid bacteria can inhibit the FXR pathway by reducing FXR target gene expression and, thus, increasing the synthesis of bile acids and maintaining the balance of cholesterol in vivo by increasing CYP7A1 expression (Ren et al., 2019). Moreover, CYP7A1 transcription is negatively regulated by FGF15 signaling. The binding of FGF15, which is secreted by the intestine, with FGFR4/β‐klotho initiates an intracellular signaling cascade involving JNK, thus inhibiting CYP7A1 transcription and bile acid synthesis (de Aguiar Vallim et al., 2013). Bobae found that *L. rhamnosus* GG treatment not only inhibited the expression of rat FXR, but also inhibited the small heterodimer chaperone SHP. SHP is a transcriptional inhibitor of FXR activation, which inhibits the transcription of CYP7A1 by inhibiting the activity of liver receptor homologues. *L. rhamnosus* GG inhibits FGF15 signaling and upregulates CYP7A1 expression in the liver, thereby reducing serum cholesterol levels (Kim et al., 2016).

### 3.3 Lactobacillus mediates the effect of NPC1L1 and ABCG5/8 on the absorption and transport of intestinal cholesterol

**FIGURE 3:**
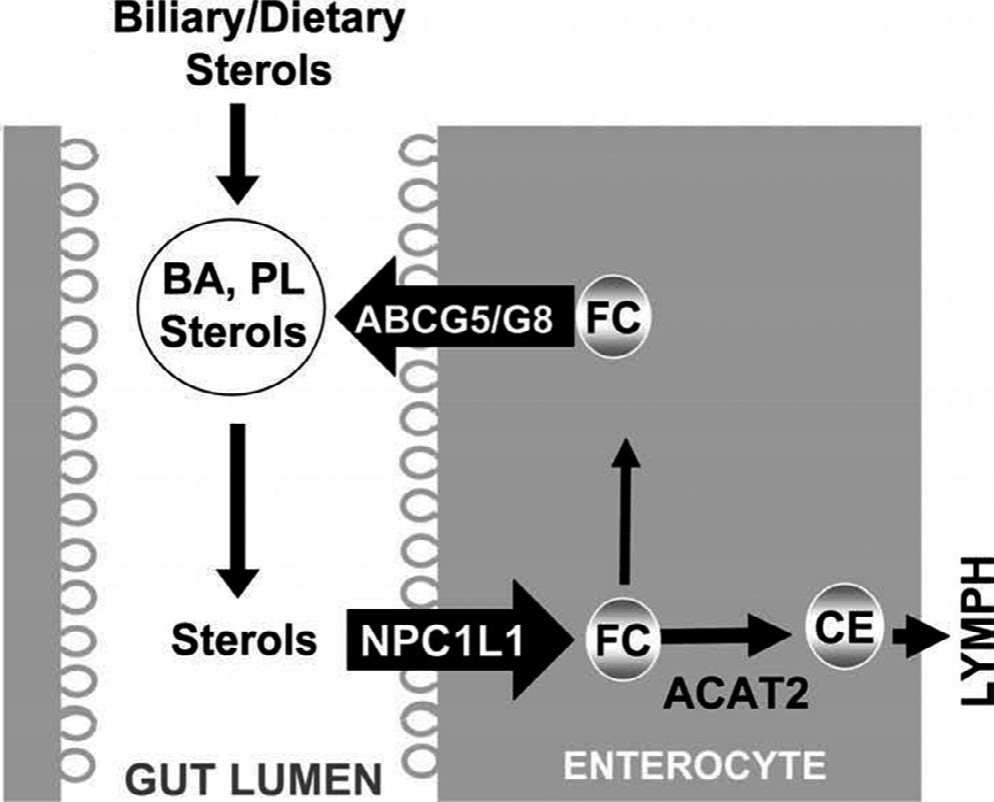
The role of NPC1L1 and ABCG5/G8 in intestinal cholesterol circulation. Biliary and dietary sterols are mixed in the gut lumen and solubilized by bile acids (BA) and phospholipids (PL) to form mixed micelles. NPC1L1 absorbs sterols, including free cholesterol (FC), from these mixed micelles at the apical surface of the enterocyte. Sterols in enterocytes can be pumped out to the gut lumen by the action of the heterodimeric ATP‐binding cassette transporters G5 and G8(ABCG5/G8) or can be esterified by acylcoenzyme A: cholesterol acyltransferase 2 (ACAT2) to yield cholesteryl ester (CE) for assembly into chylomicrons for secretion into lymph

Cholesterol absorption and excretion mainly occurs in the intestine. The intestine can absorb about 50% of dietary cholesterol every day, which is about 400 mg of dietary cholesterol (Grundy & Metzger, 1972; Wilson & Rudel, 1994); the rest is excreted through feces. The first step of intestinal cholesterol absorption is through NPC1L1, which is a unidirectional cholesterol transporter located in the brush border membrane of intestinal cells (Iyer et al., 2005) and is highly expressed in the jejunum (Wang et al., 2012). NPC1L1 regulates the transport of free cholesterol in the intestinal lumen. Cholesterol can enter intestinal epithelial cells through NPC1L1, form chylomicrons through esterification, and be transported out of cells to complete its transport in the intestine. ABCG5 and ABCG8 mainly exist in the intestine and liver of humans. They function as a heterodimer and are essential for regulating cholesterol absorption (Yu et al., 2002). Dietary cholesterol needs specific binding with ABCG5/G8 in intestinal epithelial cells to enter the intestinal lumen and complete the absorption process in the intestine (Figure 3) (Betters & Yu, 2010).

Studies have found that in the intestine, lactobacillus regulates NPC1L1 and ABCG5/G8 expression by regulating peroxisome proliferation‐activated receptors (PPAR) and liver X receptors (LXRs) and limiting cholesterol absorption by promoting the outflow of cholesterol (Repa et al., 2002; van der Veen et al., 2005). LXR is an endogenous oxysterol activated nuclear receptor and has two isoforms, namely LXRa (NR1H3) and LXRb (NR1H2). LXR regulates the expression of several genes involved in this process, such as NPC1L1, which leads to the decrease in intestinal cholesterol absorption. The activation of LXR increases ABCG5 and ABCG8 expression, thus transporting the absorbed cholesterol back to the intestinal lumen. Consistent with this finding, the administration of LXR activators can greatly reduce the absorption of net cholesterol in mice intestine.

NPC1L1 is expressed in the small intestine, most likely in the brush border membrane of intestinal epithelial cells, and is necessary for intestinal cholesterol absorption (Zhao & Dahlman‐Wright, 2010). Ying et al. monitored NPC1L1 expression to investigate the cholesterol‐lowering mechanism of *L. acidophilus* ATCC 4356 (Huang & Zheng, 2010) and found that soluble factors in the supernatant produced by ATCC 4356 in a medium containing bile acid and cholesterol could reduce cholesterol absorption by inhibiting NPC1L1 expression in Caco‐2 cells. After stimulating Caco‐2 cells with the ATCC 4356 strain, LXR expression is significantly increased in Caco‐2 cells. When interfering short RNAs depleted LXR in Caco‐2 cells, ATCC 4356 no longer reduced NPC1L1 expression, and no decrease in micellar cholesterol absorption was observed, indicating that ATCC 4356 induced NPC1L1 inhibition through the LXR pathway. ABCG5 and ABCG8 are LXR‐targeted oxysterol receptors. ABCG5/ABCG8 plays a role in the efflux of cholesterol from intestinal cells to the intestinal lumen (de Wit et al., 2008). Hong sup et al. found that metabolites of lactobacillus could not effectively promote the expression of ABCG5/8 mRNA and protein, but the cell wall treatment of these strains promoted the expression of ABCG5/8 protein and mRNA, indicating that the cell wall of lactobacillus plays a role in regulating ABCG5/8 expression (Yoon et al., 2011). Hong‐sup speculated that peptidoglycans in the bacterial cell wall are involved in ABCG5/8 activation in intestinal epithelial cells. PPARs are members of the nuclear receptor superfamily of ligand‐binding transcription factors and play an important role in lipid homeostasis by regulating the expression of lipid metabolism‐related genes. PPARs include PPARα, PPARγ, and PPARδ, of which PPARα can be activated by natural fatty acids. Moreover, PPARα has been proven to play a role in the absorption of intestinal cholesterol (Li & Chiang, 2009; Wan et al., 2013). Le et al. found that *L. plantarum* FB003 inhibits NPC1L1 mRNA expression through the nuclear receptor PPARα, which directly binds the functional PPARα response element (PPRE) upstream of the NPC1L1 gene, thereby inhibiting cholesterol uptake in Caco‐2 cells. PPARα overexpression may be a key factor in inhibiting NPC1L1 expression (Le & Yang, 2019). Ying et al. found that *L. acidophilus* ATCC 4356 inhibited NPC1L1 expression through PPARα upregulation, thereby impairing intestinal cholesterol reabsorption, and the mechanism of the intestinal reverse cholesterol transport response induced by *L. acidophilus* ATCC 4356 seems to be involves a complex crosstalk between PPARα and LXRα (Huang et al., 2014). The activation of PPAR reduces the intestinal expression of NPC1L1 and promotes reverse cholesterol transport. Thus, PPAR stimulates non‐biliary intestinal cholesterol outflow, which helps excrete neutral sterols through feces (Briand et al., 2009; Vrins et al., 2009).

### 3.4 Lactobacillus mediates the effect of SREBPs on the absorption and transport of liver cholesterol

The liver is the main organ for the synthesis and secretion of endogenous cholesterol, secreting about 1 g of cholesterol daily. Sterol regulatory element binding proteins (SREBPs) are membrane‐anchored transcription factors that are expressed in the liver. They can alter cholesterol synthesis and uptake by modulating genes encoding cholesterol biosynthetic enzymes, including HMGCR, and low‐density lipoprotein (LDL) receptors (Horton et al., 2002). The uptake of low‐density lipoprotein cholesterol (LDL‐C) in the serum is achieved through LDL receptors in the liver (Brown & Goldstein, 1983). SREBPs not only stimulate the expression of LDL receptors, but also enhance lipid synthesis (Brown & Goldstein, 1997). The mammalian SREBP gene includes three subtypes, named SREBP‐1a, SREBP‐1c, and SREBP‐2. SREBP‐1a is an effective activator of all SREBP‐responsive genes, including genes that mediate the synthesis of cholesterol, fatty acids, and triglycerides. SREBP‐1c preferentially activates the transcription of genes required for fatty acid synthesis, but not cholesterol synthesis. Similarly, SREBP‐2 has a transcription activation domain, but preferentially activates LDL receptor genes and genes required for cholesterol synthesis (Morgan et al., 2017). LDL receptor levels are regulated by the negative feedback of SREBP‐2 (Shin & Osborne, 2003). Evidence suggests a log‐linear relationship between LDL‐C concentration and relative risk of CHD. Lactobacillus can reduce serum LDL‐C (Costabile et al., 2017; Smet et al., 1995). Studies have found that lactobacillus can reduce cholesterol concentration by regulating SREBP‐2 expression (Li et al., 2014). Segawa et al. found that gavage heat‐killed *L*. *brevis* SBC8803 to C57BL/6N mice can inhibit the upregulation of SREBP‐1 and SREBP‐2 mRNA expression and reduce cholesterol accumulation (Segawa et al., 2008).

In summary, during the catabolism, absorption, and transportation of cholesterol, specific genes and key enzymes regulate cholesterol metabolism, such as the rate‐limiting enzyme HMGCR, which regulates cholesterol synthesis, and the kinase AMPK, upstream of HMGCR; CYP7A1 gene, which controls the conversion of cholesterol into bile acids; and NPC1L1 and ABCG5/G8 genes, which regulate the absorption and transport of cholesterol in the intestine and SREBP gene in the liver. Studies have shown that lactic acid bacteria can directly or indirectly regulate the expression of these genes and key enzymes.

### 3.5 Lactobacillus regulates cholesterol metabolism in vitro in cells

The in vitro cell model has been widely used. The commonly used cells include human colon adenocarcinoma cells (Caco‐2) and human liver cancer tissue cells (HepG2).

Michael et al. studied the effect of *L. plantarum* CUL66 on the uptake and metabolism of cholesterol in Caco‐2 epithelial cells. The uptake of radiolabeled cholesterol was significantly lower in Caco‐2 cells incubated with *L. plantarum* CUL66 for 5 hr than that in Caco‐2 cells not incubated with *L. plantarum* CUL66. Moreover, the *L. plantarum* CUL66‐induced expression of NPC1L1 mRNA was significantly reduced and the expression of ABCG5 and ABCG8 was increased, which indicates cholesterol efflux from the intestinal epithelium to lumen. Incubation with *L. plantarum* CUL66 for 6 hr significantly reduced cholesterol efflux from Caco‐2 cells and reduced the expression of the cholesterol transporter ABCA‐1. Moreover, in Caco‐2 cells exposed to *L. plantarum* CUL66, a significant increase in the transcription level of HMGCR was observed (Michael et al., 2016). Huang et al. studied the effects of the high BSH active lactobacillus *L. plantarum* AR113 and *L*. *casei* pWQH01 on the accumulation and metabolism of cholesterol in the HepG2 cell model induced by oleic acid and cholesterol. The results showed that both *L. plantarum* AR113 and *L*. *casei* pWQH01 significantly reduced the level of TC and the expression of HMGCR mRNA. In addition, cocultivation of the denatured HepG2 cell model with *L. plantarum* AR113 or *L*. *casei* pWQH01 significantly reduced SREBP‐1c, acetyl‐CoA carboxylase, fatty acid synthase, and TNF‐α expression and significantly increased AMPK and PPARα expression (Huang et al., 2020).

## 4 CONCLUSION

With the improvement in living standards, higher requirements for nutrition and health have emerged. The use of lactobacillus and other microorganisms to reduce cholesterol levels in the body has been attracting attention from researchers. Studies have shown that lactobacillus can reduce the cholesterol content in animals, and the mechanism of action is different.

HMGCR in liver is a rate‐limiting enzyme in the cholesterol synthesis pathway. Lactobacillus can reduce HMGCR expression by increasing AMPK phosphorylation, so as to reduce cholesterol accumulation in cells. CYP7A1 is essential for liver bile acid synthesis. Lactobacillus can regulate CYP7A1 expression through FXR and LXRα pathways. At the same time, lactobacillus regulates genes encoding cholesterol biosynthetic enzyme, including HMGCR, and LDL‐R through SREBP to change the synthesis and uptake of cholesterol in the liver.

ABCG5 and ABCG8, expressed in the small intestine and liver of humans, regulate cholesterol transport. LXR can activate the expression of genes involved in cholesterol efflux, including ABCA1 and ABCG5/8. Lactobacillus can upregulate ABCG5/8 protein expression by activating LXR expression. NPC1L1 mediates cholesterol absorption by transporting cholesterol across the membrane into small intestinal cells. Lactobacillus can inhibit NPC1L1 mRNA expression through the nuclear receptor PPARα.

In summary, lactobacillus can regulate the cholesterol content in the body by controlling the expression of related genes to regulate cholesterol metabolism.

## 5 DISCUSSION

The effect of lactobacillus on reducing the cholesterol content in the mouse obesity model has been confirmed in previous studies. Clinical studies have shown that different lactobacillus strains can not only reduce fat accumulation and affect the metabolism of cholesterol and triglycerides, but also regulate the intestinal microbiota and affect non‐alcoholic fatty liver disease (NAFLD). Excessive cholesterol in the body will increase the number of Gram‐positive *Firmicutes* and decrease the number of Gram‐negative *Bacteroides* in the intestine. The intervention of lactic acid bacteria can increase the number of lactobacillus and Bifidobacterium and normalize the intestinal microbiota. NAFLD is defined as excessive fat deposition in hepatocytes other than alcohol and other clear liver damage factors; clinical studies have shown that intake of lactobacillus can improve non‐alcoholic steatosis by lowering cholesterol and morphological structure of the liver. Therefore, intervention and regulation by lactobacillus is a promising treatment and prevention method for hypercholesterolemia.

## AUTHORS CONTRIBUTION

**Kaihui Cao:** Conceptualization (lead); Formal analysis (lead); Writing‐original draft (lead); Writing‐review & editing (lead). **KaiPing Zhang:** Writing‐review & editing (lead). **Jianjun Tian:** Funding acquisition (lead); Writing‐review & editing (lead). **JunJie Ma:** Writing‐review & editing (equal). **MuRan Ma:** Writing‐review & editing (equal). **Ye Jin:** Writing‐review & editing (equal).

## Data Availability

The data of this study are openly available.
